# Serum GDF-8 levels change dynamically during controlled ovarian hyperstimulation in patients undergoing IVF/ICSI-ET

**DOI:** 10.1038/srep28036

**Published:** 2016-06-22

**Authors:** Lanlan Fang, Yiping Yu, Ruizhe Zhang, Jingyan He, Ying-Pu Sun

**Affiliations:** 1 Reproductive Medical Center, The First Affiliated Hospital of Zhengzhou University, Zhengzhou 450052, China

## Abstract

Growth differentiation factor-8 (GDF-8) is found in the human serum, follicular fluid and granulosa cells. Our previous studies have shown that the human cumulus expansion and steroidogenesis can be regulated by GDF-8. However, thus far, the expression profile of GDF-8 in serum and whether the level of serum GDF-8 influences pregnancy results for patients treated with *in vitro* fertilization/intracytoplasmic sperm injection-embryo transfer (IVF/ICSI-ET) is totally unknown. In this study, we showed that GDF-8 had a dynamic trend during controlled ovarian hyperstimulation (COH) procedure. On human chorionic gonadotropin (hCG) administration day, patients with a GDF-8 level higher than 4.7 ng/ml had lower progesterone levels and a higher pregnancy rate. From hCG day to oocyte pick-up day, patients with a GDF-8 decrease greater than 1.3 ng/ml had a higher progesterone increase and a higher pregnancy rate. Importantly, the levels of GDF-8 were negatively correlated with progesterone levels. Our findings provide evidences that GDF-8 plays an important role in ensuring successful pregnancy by regulating progesterone levels.

Effective prediction and improvement of clinical pregnancy results have been the most important issues for many decades since the application of *in vitro* fertilization/intracytoplasmic sperm injection-embryo transfer (IVF/ICSI-ET) for infertile patients. Many studies have reported that there are several useful clinical indexes for predicting pregnancy outcome in patients treated with IVF/ICSI-ET, such as age, antral follicle count (AFC), serum hormone levels, retrieved oocytes and endometrium receptivity^1^,^2^,^3^,^4^,^5^. Younger women with a higher AFC, more retrieved oocytes, and higher endometrium receptivity may have better pregnancy results^1^,^2^,^3^,^4^,^5^.

Despite the above indexes, other studies have indicated that many growth factors play important roles in the prediction of ovarian response and pregnancy results. Transforming growth factor-β (TGF-β) superfamily members (TGF-βs, bone morphogenetic proteins (BMPs), growth differentiation factors (GDFs), antimüllerian hormone (AMH), activins and inhibins) are widely expressed in the ovary and participate in various aspects of female reproduction^6^. The expression level of TGF-β1 is reported to be significantly higher in the follicular fluid of pregnant women than non-pregnant women^7^,^8^. Serum AMH has recently been proved to be closely associated with ovarian response, retrieved oocyte number and clinical pregnancy rates^9^,^10^. Patients with the greatest AMH decline during controlled ovarian hyperstimulation (COH) have more retrieved oocytes and a higher clinical pregnancy rate^10^.

Growth differentiation factor-8 (GDF-8), another important member of the TGF-β superfamily, also known as myostatin, was initially identified to be synthesized and secreted by muscle cells^11^. It has been well characterized that GDF-8 is a critical autocrine/paracrine inhibitor of skeletal muscle growth and differentiation^11^,^12^,^13^. Interestingly, GDF-8 is also expressed in the human reproductive system, such as in granulosa cells, follicular fluid and trophoblasts^14^,^15^,^16^,^17^. In addition, recently, our group has revealed some novel biological functions of GDF-8 in regulation of human cumulus expansion and steroidogenesis^15^,^16^. Our previous studies show that GDF-8 down-regulates pentraxin 3 and steroidogenic acute regulatory protein (StAR) expression, whereas it increases P450 aromatase expression in human granulosa cells^15^,^16^,^18^. The wide expression pattern and functions of GDF-8 in the reproductive system indicate its potential significance in the regulation of female reproductive activities, especially in steroidogenesis, which is tightly associated with pregnancy results. However, most of the above studies were focused on the *in vitro* granulosa cell model. The *in vivo* function of GDF-8 in female reproduction is still unknown.

As we all know, serum hormone levels, especially estradiol (E2) and progesterone (P4) levels, vary during the process of COH and influence pregnancy outcome for patients treated with IVF/ICSI-ET^1^,^2^,^19^. In addition, recently, we have demonstrated that GDF-8 increases the E2 levels while it decreases P4 levels in human granulosa cells^15^,^18^. However, the relationship between serum GDF-8 levels and pregnancy results remains completely unknown. In the present study, for the first time, we revealed that the serum GDF-8 protein levels dynamically changed during the process of COH. Serum GDF-8 level was a valuable predictor for pregnancy for patients treated with IVF/ICSI-ET. Before human chorionic gonadotrophin (hCG) administration, higher levels of GDF-8 might be beneficial for pregnancy by keeping a lower P4 level in serum; however, after hCG administration, lower levels of GDF-8 might be crucial for early embryo implantation by maintaining the high levels of P4.

## Results

### GDF-8 levels in serum dynamically change during the process of COH

**Figure 1:**
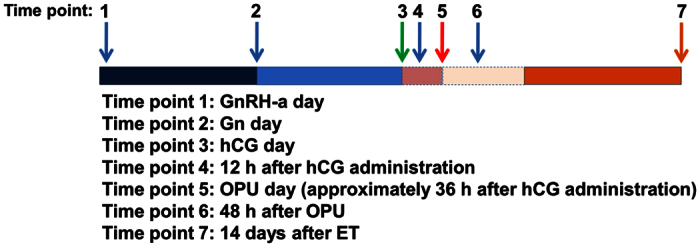
The flow chart of sample collection. Blood samples were obtained by venipuncture at seven time points during COH: Time point 1 (GnRH-a day): initiate Gonadotropin-releasing hormone agonist (GnRH-a) administration day; Time point 2 (Gn day): initiate Gonadotropin (Gn) administration day; Time point 3 (hCG day): human chorionic gonadotropin (hCG) administration day (the serum was collected before hCG injection); Time point 4: 12 h after hCG administration; Time point 5 (OPU day): oocyte picked-up (OPU) day (about 36 h after hCG administration); Time point 6: 48 h after OPU and Time point 7: 14 days after embryo transfer (ET).

**Figure 2:**
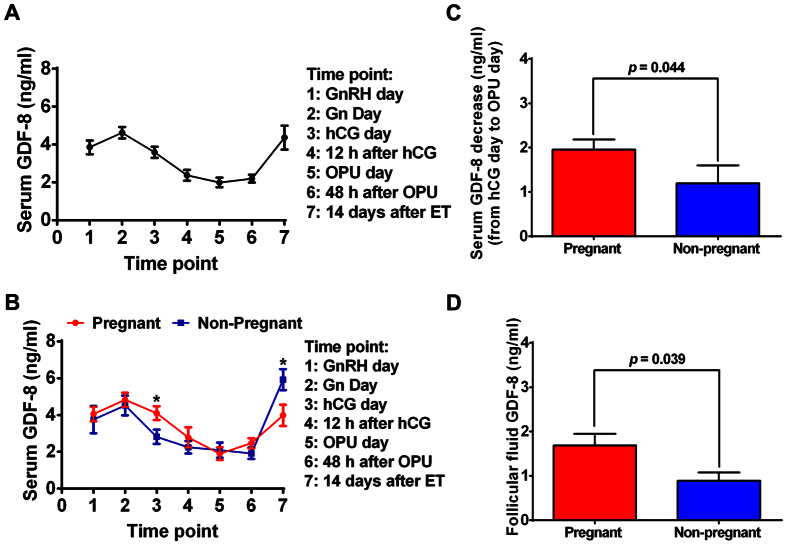
The expression levels of serum GDF-8 during the process of COH and the relationship between serum GDF-8 level and pregnancy outcome. (**A**) Serum samples at different time points were obtained from 19 patients undergoing COH treatment. The level of GDF-8 in serum was measured by ELISA. (**B**) The 19 patients were divided into 2 groups according to their pregnancy results. The serum levels of GDF-8 at different time points of pregnant group (n = 11, labeled with red color) and non-pregnant group (n = 8 labeled with blue color) were demonstrated. (**C**,**D**) The 19 patients were divided into 2 groups according to their pregnancy results. The decreases in serum GDF-8 levels from hCG day to OPU day (**C**) and the GDF-8 levels in follicular fluid (**D**) were demonstrated. The results are expressed as the mean ± SEM. *p < 0.05 compared between pregnant group and non-pregnant group at the same time point.

Although many studies have focused on the role of GDF-8 in the regulation of reproductive functions^14^,^15^,^16^,^17^,^18^,^20^, none of them have examined the endogenous GDF-8 secretion pattern during the process of COH. We hypothesized that serum GDF-8 levels may vary during the process of COH. Thus, we collected blood samples from patients undergoing COH at seven different time points: *Time point* 1, GnRH-a day; *Time point* 2, Gn day; *Time point* 3, hCG day; *Time point* 4, 12 h after hCG administration; *Time point* 5, OPU day; *Time point* 6, 48 h after OPU; and *Time point* 7: 14 days after ET (Fig. 1). As shown in Fig. 2A, GDF-8 level in serum increased slightly after GnRH-a administration; however, it decreased greatly after Gn injection, especially at 12 h after hCG administration. Interestingly, serum GDF-8 level was primarily down-regulated in the early luteal phase, and then it was up-regulated in the late luteal phase.

### The relationship between serum GDF-8 levels and pregnancy outcome

To analyze the relationship between GDF-8 levels and pregnancy results, we next compared the GDF-8 levels in serum at different time points and in follicular fluid between the pregnant and non-pregnant groups. Our results showed that the GDF-8 level variation trends were similar in the pregnant and non-pregnant groups. However, there were significant differences in GDF-8 levels between the two groups on hCG day and at 14 days after ET (Fig. 2B). The GDF-8 level in serum on hCG day was significantly higher in the pregnant group than in the non-pregnant group (4.1 ± 0.4 *vs* 3.0 ± 0.4, *p* = *0.038*). However, the level of GDF-8 in serum 14 days after ET was significantly lower in the pregnant group than in the non-pregnant group (4.0 ± 0.6 *vs* 5.9 ± 0.9, *p* = *0.048*). In addition, our results showed that decreases in serum GDF-8 levels from hCG day to OPU day were more pronounced in the pregnant group than in the non-pregnant group (1.9 ± 0.2 vs 1.1 ± 0.4, *p* = 0.044, Fig. 2C). Because follicular fluid is the important environment for the regulation of folliculogenesis and steroidogenesis, we also measured the GDF-8 levels in follicle fluid, and our results showed that GDF-8 level in follicular fluid collected at the time of OPU was significantly higher in the pregnant group than in the non-pregnant group (1.6 ± 0.3 *vs* 0.9 ± 0.2, *p* = *0.039*, Fig. 2D). Interestingly, the P4 increase was higher in the pregnant group than in the non-pregnant group (23.6 ± 3.1 *vs* 18.5 ± 5.2 *p* = *0.391*) although it did not reach the statistical difference (Supplemental Table 1). Age, BMI, AFC, basal FSH, luteinizing hormone (LH), E2, P4 and prolactin (PRL), the total duration and amount of Gn administered, P4 and E2 in serum on hCG day and on OPU day, and the number of retrieved oocytes showed no differences between the two groups (Supplemental Table 1).

### Serum GDF-8 level on hCG day and a decrease in GDF-8 level from hCG day to OPU day are valuable predictors of pregnancy outcome

**Figure 3:**
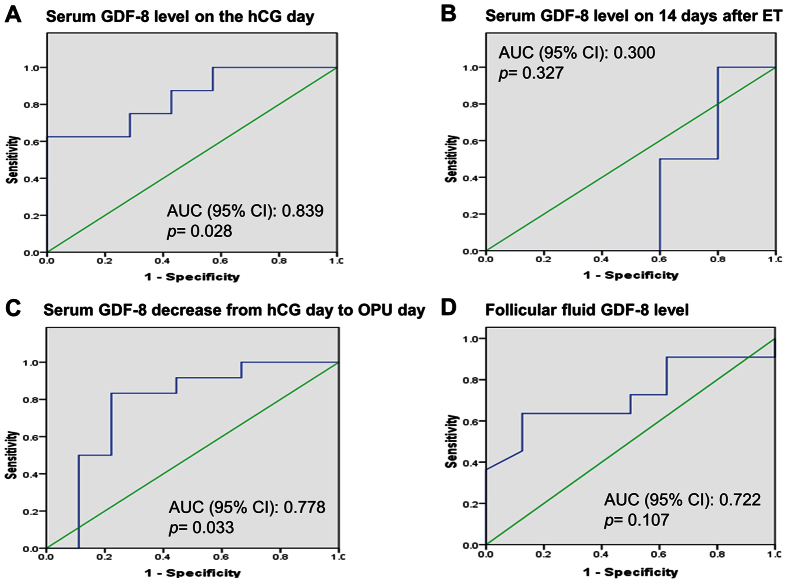
Receiver operating characteristic (ROC) curve analysis. The ROC curve for serum GDF-8 level on hCG day (**A**), serum GDF-8 level 14 days after ET (**B**) serum GDF-8 decrease from hCG day to OPU day (**C**) and follicular fluid GDF-8 level in predicting pregnancy were analyzed. Diagonal segments are produced by ties.

The above data indicated that GDF-8 level was related with pregnancy results, so we next analyzed the predictive value of GDF-8 for the pregnancy. The area under the curve (AUC) of GDF-8 level in serum on hCG day for predicting pregnancy was 0.839 (Fig. 3A, *p* = *0.028*). The AUC of GDF-8 level in serum 14 days after ET for predicting pregnancy was 0.300 (Fig. 3B, *p* = *0.327*). The AUC of a GDF-8 level decrease from hCG day to OPU day in serum for predicting pregnancy was 0.778 (Fig. 3C, *p* = *0.033*). The AUC of GDF-8 level in follicular fluid for predicting pregnancy was 0.722 (Fig. 3D, *p* = *0.107*). These results indicated that the serum GDF-8 level on hCG day and a GDF-8 decrease from hCG day to OPU day were pivotal predictors for the pregnancy outcome. Furthermore, we analyzed the sensitivity, specificity, positive predictive value (PPV), negative predictive value (NPV) and best cutoff value for GDF-8 level in serum on hCG day and for the GDF-8 decrease. Our data showed that GDF-8 with a value of 4.7 ng/ml on hCG day predicted pregnancy with sensitivity of 62.5%, specificity of 100%, PPV of 100 and NPV of 57.2, whereas a GDF-8 decrease from hCG day to OPU day in serum with a value of 1.3 ng/ml predicted pregnancy with sensitivity of 83.3%, specificity of 77.8%, PPV of 81.8 and NPV of 75 (Supplemental Table 2).

### Higher level of GDF-8 with lower P4 level in serum on hCG day is associated with a significantly higher pregnancy rate

**Figure 4:**
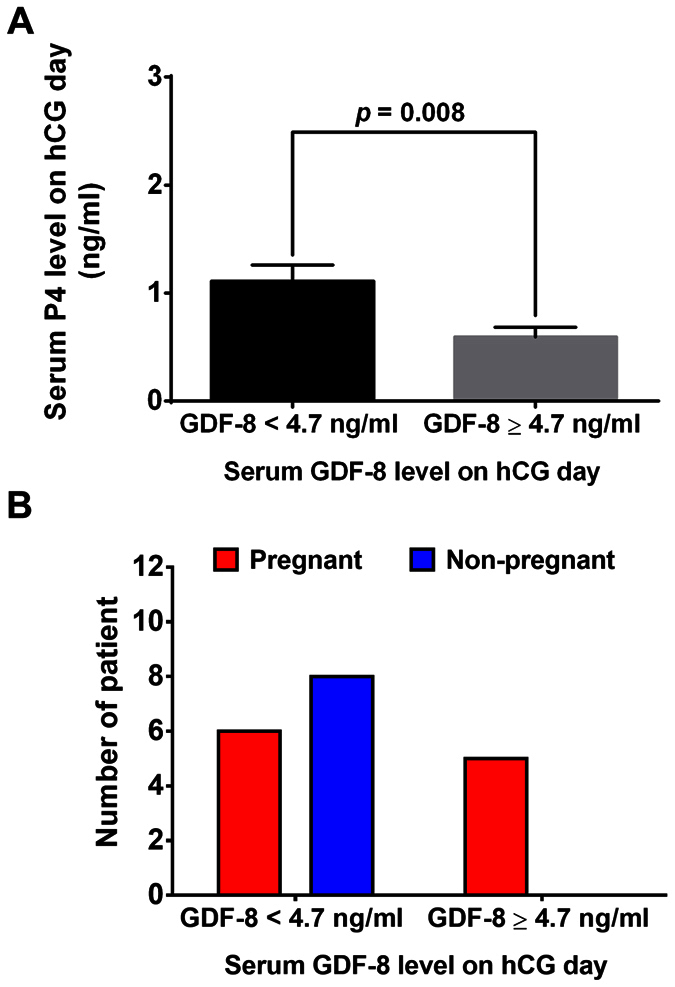
The relationship of serum GDF-8 with P4 level and pregnancy results on hCG day. (**A**) Patients were divided into two groups according to the cut-off GDF-8 level in serum on hCG day (GDF-8 < 4.7 ng/ml *vs* GDF-8 ≥ 4.7 ng/ml). Serum P4 level was measured by ECLIA. B, Patients were divided into two groups according to the cut-off GDF-8 value in serum on hCG day (GDF-8 < 4.7 ng/ml *vs* of GDF-8 ≥ 4.7 ng/ml). The pregnancy rate was calculated and compared between the two groups. The results are expressed as the mean ± SEM or number.

In addition, we divided the patients into two groups according to the cut-off GDF-8 level in serum on hCG day (GDF-8 < 4.7 ng/ml *vs* GDF-8 ≥ 4.7 ng/ml) and compared the general characteristics of these two groups. No differences were found between the two groups in terms of age, BMI, AFC, basal FSH, LH, E2, P4 and PRL, total duration and amount of Gn administered, E2 in serum on hCG day, P4 and E2 in serum on OPU day, or in retrieved oocytes (Supplemental Table 3). However, P4 in serum on hCG day was significantly higher in group of GDF-8 < 4.7 ng/ml than in group of GDF-8 ≥ 4.7 ng/ml (Fig. 4A). In addition, as expected, the pregnancy rate in group of GDF-8 < 4.7 ng/ml was significantly lower than that in group of GDF-8 ≥ 4.7 ng/ml (42.9% *vs* 100.0%, *p* = *0.025*, Fig. 4B). These results indicated that higher GDF-8 level with a lower P4 level in serum on hCG day was associated with a significantly higher pregnancy rate.

### A greater decrease in GDF-8 level with a higher P4 increase in serum from hCG day to OPU day is associated with a significantly higher pregnancy rate

**Figure 5:**
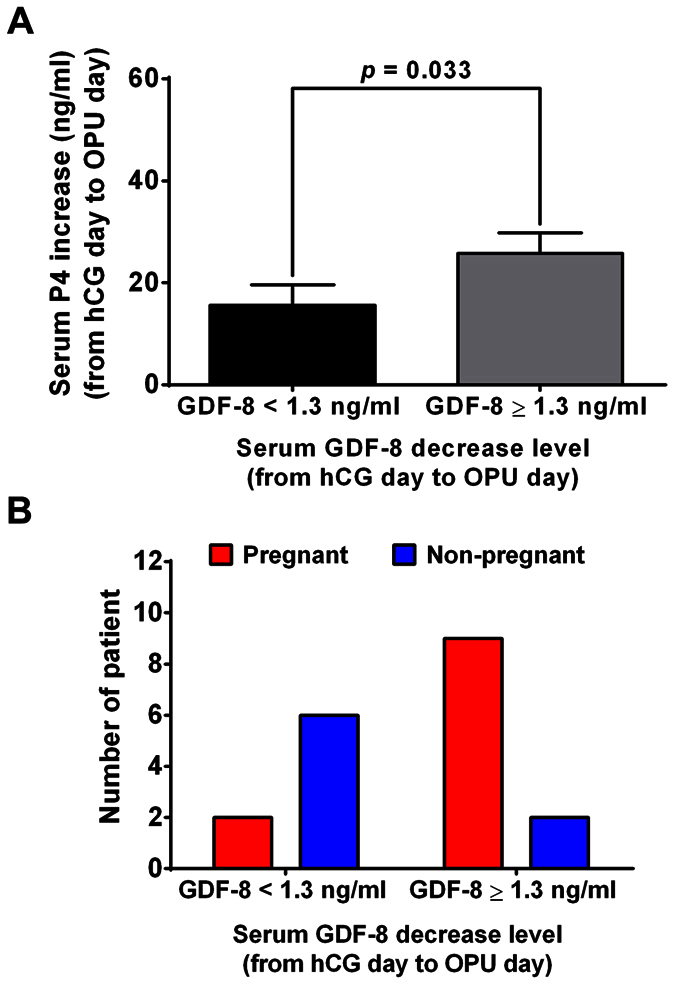
The relationship of serum GDF-8 with P4 level changes and pregnancy results from hCG day to OPU day. (**A**) Patients were divided into two groups according to the cut-off level for GDF-8 decrease in serum from hCG day to OPU day (GDF-8 decrease <1.3 ng/ml *vs* GDF-8 decrease ≥1.3 ng/ml). Serum P4 level was measured by ECLIA in the two groups. B, Patients were divided into two groups according to the cut-off levels for GDF-8 decrease in serum from hCG day to OPU day (GDF-8 decrease <1.3 ng/ml *vs* GDF-8 decrease ≥1.3 ng/ml). The pregnancy rate was calculated and compared between the two groups. The results are expressed as the mean ± SEM or number.

As mentioned above, the GDF-8 level decrease in serum from hCG day to OPU day was found to be significantly different between the pregnant and non-pregnant groups. To examine the effect of the GDF-8 level decrease on general patient characteristics, we divided the patients into two groups according to the cut-off value of the GDF-8 level decrease in serum from hCG day to OPU day (GDF-8 < 1.3 ng/ml *vs* GDF-8 ≥ 1.3 ng/ml) and compared the general characteristics of these two groups. No differences were found between the two groups in terms of age, BMI, AFC, basal FSH, LH, E2, P4 and PRL, total duration and amount of Gn administered, E2 in serum on hCG day, P4 and E2 in serum on OPU day, or retrieved oocytes (Supplemental Table 4). However, the P4 increase in serum from hCG day to OPU day in group of GDF-8 < 1.3 ng/ml was significantly lower than that in group of GDF-8 ≥ 1.3 ng/ml (Fig. 5A). In addition, the pregnancy rate in group of GDF-8 < 1.3 ng/ml was significantly lower than that in group of GDF-8 ≥ 1.3 ng/ml (25.0% *vs* 81.8%, *p* = *0.013*, Fig. 5B). These results indicated that a greater GDF-8 decrease with a higher P4 increase in serum from hCG day to OPU day was associated with a significantly higher pregnancy rate.

### GDF-8 levels are negatively correlated with the levels of P4

**Figure 6:**
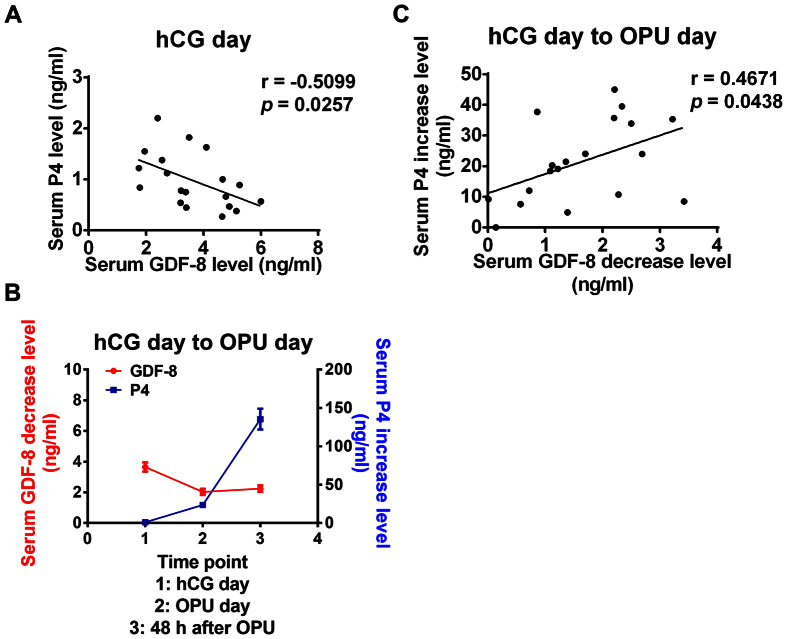
The relationship between serum GDF-8 level and P4 level. (**A**) GDF-8 level was negatively correlated with P4 level in serum on hCG day. (**B**) The decrease of GDF-8 level was accompanied by the increase of P4 level in serum at the time points as indicated. The results are expressed as the mean ± SEM. (**C**) From hCG day to OPU day, the decrease of GDF-8 was positively correlated with the increase of P4 level in serum.

Our previous study has demonstrated that GDF-8 can down-regulate StAR expression and decrease P4 production in cultured human granulosa cells and the level of GDF-8 is negatively correlated with P4 level in human follicular fluid^18^. Therefore, we next examined the correlation of GDF-8 and P4 level in serum on hCG day. Our results showed that GDF-8 level was negatively correlated with level of P4 in serum on hCG day (Fig. 6A). In addition, we found that the GDF-8 level decrease was accompanied by a P4 increase from hCG day to OPU day as well as at 48 h after OPU (Fig. 6B). Moreover, the GDF-8 level decrease was positively correlated with the P4 increase in serum from hCG day to OPU day (Fig. 6C). Taken together, these results indicate that after hCG administration, the decrease of GDF-8 was very important for maintaining the high level of P4.

## Discussion

Hypothalamus-pituitary-ovary axis (HPOA) is the core regulation system of folliculogenesis and steroidogenesis. However, many paracrine and autocrine growth factors also participate in this process and play significant roles. Our previous studies have revealed the functions of GDF-8 in the regulation of follicular growth and hormone production. Additionally, in this study, we firstly showed that there was a dynamic trend in GDF-8 levels in the process of COH in patients treated with IVF/ICSI-ET. The GDF-8 level rose slightly after GnRH-a administration. However, it decreased greatly after Gn injection, especially at 12 h after hCG administration. More interestingly, GDF-8 level was primarily down-regulated in the early luteal phase, then up-regulated in the late luteal phase. COH is a complex process accompanied by continuous changes in folliculogenesis and steroidogenesis. The dynamic balance of steroidogenesis is the key regulator of normal follicular growth. This study further demonstrated the functions of GDF-8 in the regulation of folliculogenesis and steroidogenesis.

Considering that pregnancy results are affected by many external factors, we adjusted the general characteristics of the infertile patients (Supplemental Table 1). All the included patients were within the normal range of age, BMI, and basal hormone levels and were treated with the standard pituitary down-regulation long protocol. Our results showed an association between GDF-8 level changes during the process of COH and pregnancy results. This observation indicated that a dynamic change of GDF-8 levels during COH was predictive of IVF/ICSI-ET pregnancy outcome independent of age, AFC, ovarian response and Gn dose. Statistically significant associations were observed between pregnancy and serum GDF-8 level on hCG day, the GDF-8 level decrease from hCG day to OPU day, and GDF-8 level at 14 days after ET. The GDF-8 levels in follicular fluid and pregnancy results were also observed to be significantly correlated. ROC curve analysis showed that GDF-8 level on hCG day (with a cutoff point of 4.7 ng/ml) and GDF-8 level decrease from hCG day to OPU day (with a cutoff point of 1.3 ng/ml) had higher predictive potential for pregnancy than other time points (Supplemental Table 2). These results suggested that the level of GDF-8 on hCG day and the GDF-8 level decrease from hCG day to OPU day might be used as an indicator for evaluating pregnancy outcomes in patients treated with IVF/ICSI-ET. In the present study, our results showed a significant correlation between serum GDF-8 level and pregnancy outcomes. However, we are aware that the number of patients enrolled in the current study was limited. Thus, future studies with large sample size will be needed to further evaluate the relationship between serum GDF-8 level and pregnancy rate in patients treated with IVF/ICSI-ET.

Although the dynamic trend and predictive value of GDF-8 level for pregnancy results have been revealed in the present study, the underlying mechanism remains unclear. Future investigation of the mechanism will be needed to guide new clinical treatment strategies. Premature elevation of P4 on hCG day has been shown to reduce the embryo implantation rate, pregnancy rate and live birth rate^2^,^21^. For poor responder patients, premature P4 > 0.9 ng/ml led to drastically reduced pregnancy rates^22^. Our previous study shows that GDF-8 down-regulates StAR expression and decreases P4 production in human granulosa cells^18^. This finding clearly indicates that GDF-8 plays a critical role in the regulation of P4 production which may subsequently prevent the premature luteinization before hCG administration and ensure the successful pregnancy. Our data in this study showed that on the hCG day the P4 level was lower in patients with a higher serum GDF-8 level, whereas it was significantly higher in patients with a lower serum GDF-8 level. Furthermore, GDF-8 level was negatively correlated with the level of P4 in serum on hCG day. Taken together, these results all clearly indicate that the higher GDF-8 level in serum may be a protective factor before hCG administration, which leads to a higher pregnancy rate by preventing premature P4 elevation.

It is well known that, after the LH surge, luteinized granulosa cells are stimulated to produce abundant P4 23,24. The normally elevated P4 levels after the LH surge are critical for the regulation of luteinization and to maintain a successful pregnancy at the early implantation stage. Deficient secretion of P4 in the luteal phase results in early spontaneous abortion^25^. However, to date, only a small number of studies have focused on the P4 level in serum after hCG injection^26^. It has been shown that elevated P4 on OPU day is associated with significantly lower implantation and ongoing pregnancy rates^27^. To provide more clinically useful information on whether embryos should be frozen and transferred, P4 level measurement should be considered on hCG day and also the day after hCG administration^26^. Despite this significant possibility, the changes of P4 level after hCG administration has never been described. In the present study, our results showed that the P4 increase was higher in the pregnant group than in the non-pregnant group, although these results did not reach statistical significance. These results may be due to the small sample size. More importantly, a greater decrease in GDF-8 with a higher P4 increase in serum from hCG day to OPU day was associated with a significantly higher pregnancy rate. These results indicate that after hCG administration, a decrease in GDF-8 played a delicate role in maintaining the high level of P4.

In summary, this study, for the first time, revealed that the GDF-8 levels dynamically changed during the process of COH. Serum level of GDF-8 on the hCG day and the level of GDF-8 decrease from hCG day to OPU day were valuable predictors of pregnancy during IVF/ICSI-ET treatment. In addition, our results suggest that before hCG administration, higher GDF-8 may be beneficial factor for pregnancy by keeping a lower P4 level in serum. After hCG administration, decrease of GDF-8 level may be critical for early embryo implantation and successful pregnancy by maintaining the high level of P4. This study provides evidences that GDF-8 plays an important role in ensuring successful pregnancy by regulating progesterone levels.

## Materials and Methods

### Patients

Our prospective study included human serum and follicular fluid samples obtained from 19 infertile women undergoing IVF/ICSI-ET cycles in this study. These patients were treated at the Reproductive Medicine Center at the First Affiliated Hospital of Zhengzhou University in China from January 2015 to May 2015. Informed consent was obtained from all patients. The study received approval and was carried out in accordance with the approved guidelines from the Zhengzhou University Research Ethics Board. For all the patients, the inclusion criteria were: 1) age between 20 and 35; 2) BMI between 19 and 24.9; 3) regular menstrual cycles; 4) tubal factor infertility or male factor fertility; and 5) patients without any complications, such as diabetes or abnormal thyroid function. The exclusion criteria were: 1) patients with polycystic ovarian syndrome (PCOS), endometriosis or diminished ovarian reserve; 2) patients with chromosome abnormality; and 3) patients with hydrosalpinx. Our preliminary experiment indicated that the average GDF-8 level in the follicular fluid of the pregnant patients was 1.6 ± 0.3 ng/ml (n = 3) compared to a level of 0.8 ± 0.3 ng/ml (n = 3) in the non-pregnant patients. The pregnancy rate is about 60% in our reproductive center. These results indicate that to detect a difference in GDF-8 level between the two groups with α = 0.05 and β with a power of 90%, we needed at least 10 patients in the pregnant group and another 7 patients in the control group. We therefore chose 19 patients, including 11 pregnant patients and 8 non-pregnant patients. The clinical characteristics of these 19 patients were all in the normal range and summarized in Supplemental Table 1.

### COH protocol

All of the patients were treated with the standard long protocol. At the mid-luteal phase, the 0.1 mg gonadotropin-releasing hormone agonist (GnRH-a), triptorelin (Ipsen Pharma Biotech, Boulogne-Billancourt, France) was administered s.c. daily. The initial GnRH-a administration day was defined as GnRH-a day. Approximately 14 days after injection of the GnRH-a, when desensitization was achieved, gonadotropin (Gn) and recombinant follicle-stimulating hormone (FSH) (Gonal-F; Merck, Darmstadt, Germany) at a dosage of 150–300 IU was administered daily. The initial Gn administration day was defined as Gn day. When at least three follicles had reached 18 mm, hCG (Livzon, Guangdong, China) was injected. The hCG administration day was defined as hCG day. Oocyte pick-up (OPU) was scheduled approximately 34–36 h after hCG injection by transvaginal ultrasound-guided follicular aspiration, and the day was defined as OPU day. Progesterone in oil was used for luteal support at a dose of 60 mg per day after OPU. One or two embryos of the highest quality were transferred to the uterine cavity at the third or fifth day after oocyte retrieval. Biochemical pregnancy was diagnosed by serum β-hCG 14 days after embryo transfer (ET), and this day was defined as 14 days after ET. Clinical pregnancy was defined as identification of a gestational sac 35 days after the ET.

### Collection of blood and follicular fluid

Blood samples were obtained by venipuncture at seven time points during COH (Supplemental Fig. 1:*Time point* 1, GnRH-a day; *Time point* 2, Gn day; *Time point* 3, hCG day; *Time point* 4, 12 h after hCG administration; *Time point* 5, OPU day, approximately 36 h after hCG administration; *Time point* 6, 48 h after OPU; *Time point* 7, 14 days after ET). On Time point 1, 2 and 3, serum was collected at approximately 08:00 am, before the GnRH, Gn and hCG administration. On Time point 4, serum was collected at approximately 12 h after the hCG injection. On Time point 5, 6 and 7, serum was collected at approximately 08:00 am on the defined day. After collection, the serum was stored at −80 °C until it was assayed. Follicular fluid was collected when the oocytes were retrieved. Only the first follicular fluid aspirate without blood or flushing solution was used for analysis. After 10 minutes of centrifugation at 1200 rpm, the supernatant was stored at −80 °C until it was assayed.

### ELISA and ECLIA

Human GDF-8 ELISA kits (R&D Systems) were used in accordance with the manufacturer’s protocol. The serum and human follicular fluids were collected, and the GDF-8 levels in the serum and human follicular fluids were measured by ELISA. P4 and E2 levels in the serum were measured by electrochemiluminescence immunoassay (ECLIA). P4 and E2 ECLIA kits (Roche Diagnostics, Germany) were used in accordance with the manufacturer’s protocol.

### Statistical analysis

The results are presented as the means or means ± SEM. The independent sample t-test was used for the comparison between two groups. A receiver operator characteristic (ROC) curve was used to test the discriminatory power of GDF-8 for pregnancy. A significant difference was defined as *p* < 0.05.

## Additional Information

**How to cite this article**: Fang, L. *et al.* Serum GDF-8 levels change dynamically during controlled ovarian hyperstimulation in patients undergoing IVF/ICSI-ET. *Sci. Rep.* **6**, 28036; doi: 10.1038/srep28036 (2016).

## Supplementary Material

Supplementary Information
