# Supplementary material for: Serum GDF-8 levels change dynamically during controlled ovarian hyperstimulation in patients undergoing IVF/ICSI-ET
Source: Sci Rep. 2016 Jun 22;6:28036. doi: 10.1038/srep28036 (PMC4916440; doi:10.1038/srep28036)
Supplement: Supplementary Information [file srep28036-s1.pdf]

Serum GDF-8 levels change dynamically during controlled ovarian hyperstimulation in patients undergoing IVF/ICSI-ET

Lanlan Fang, Yiping Yu, Ruizhe Zhang, Jingyan He, Ying-Pu Sun[*]
Reproductive Medical Center, The First Affiliated Hospital of Zhengzhou University, Zhengzhou, China 450052
[*]Corresponding author

Supplementary Table 1

	Pregnant Group (n=11)	Non-pregnant Group (n=8)
AGE	29.8±1.7	30.0±1.0
BMI	21.6±0.8	23.9±1.8
Number of AFC	12.7±1.5	14.9±1.6
Basle FSH (mIU/ml)	6.7±0.5	6.3±0.5
Basle LH (mIU/ml)	5.4±0.5	4.3±0.7
Basle E2 (pg/ml)	32.5±3.4	68.8±18.3
Basle P4 (ng/ml)	0.6±0.1	0.6±0.1
Basle T (pg/ml)	0.2±0.03	0.2±0.05
Basle PRL (ng/ml)	19.6±2.4	21.9±4.0
Total Gn days	11.0±0.7	11.0±0.7
Total Gn dose (ampoules)	1904.5±249.5	1500.0±133.0
Serum E2 on hCG day (pg/ml)	4628.8±644.2	5427.8±683.2
Serum P4 on hCG day (ng/ml)	0.9±0.2	1.0±0.2
Serum E2 on OPU day (pg/ml)	2591.5±402.8	3575.4±749.0
Serum P4 on OPU day (ng/ml)	24.6±3.5	22.0±5.7
P4 increase from hCG day to OPU day (ng/ml)	23.6±3.1	18.5±5.2
Number of retrieved oocyte	12.5±1.8	12.8±1.4

BMI, body mass index; AFC, antral follicle count; FSH, follicle stimulating hormone; LH, luteinizing hormone; E2, estradiol; P4, progesterone; T, testosterone; PRL, prolactin; Gn: gonadotropin; hCG: human chorionic gonadotropin; OPU: oocyte picked-up

Data are presented as mean ± SEM

Supplementary Table 2

	Serum GDF-8 on hCG day	GDF-8 decrease from hCG day to OPU day
AUC (95% CI)	0.839	0.778
p value	0.028	0.033
Best cutoff point (ng/ml)	4.7	1.3
Sensitivity (%)	62.5	83.3
Specificity (%)	100	77.8
PPV (%)	100	81.8
NPV (%)	57.2	75

AUC: area under the curve; PPV: positive predictive value; NPV: negative predictive value

Supplementary Table 3

	GDF-8 < 4.7 ng/ml (n=14)	GDF-8 ≥ 4.7 ng/ml (n=5)
AGE	30.4±0.9	26.4±3.0
BMI	22.9±1.1	21.1±1.1
Number of AFC	13.8±1.2	13.0±2.8
Basle FSH (mIU/ml)	6.1±0.4	7.3±0.7
Basle LH (mIU/ml)	4.3±0.4	6.1±0.7
Basle E2 (pg/ml)	54.1±16.6	23.4±3.7
Basle P4 (ng/ml)	0.6±0.1	0.4±0.07
Basle T (ng/ml)	0.2±0.04	0.2±0.01
Basle PRL (ng/ml)	21.0±2.7	18.7±4.0
Total Gn days	11.2±0.6	10.0±0.9
Total Gn dose (Ampoules)	1783.0±145.9	1597.5±491.9
Serum E2 on hCG day (pg/ml)	5207.4±559.5	4287.0±906.3
Serum P4 on hCG day (ng/ml)	1.1±0.2	0.6±0.1 *
Serum E2 on OPU day (pg/ml)	3082.1±479.1	2693.2±613.9
Serum P4 on OPU day (ng/ml)	22.4±3.7	26.5±4.7
Number of retrieved oocyte	12.1±1.1	13.8±1.9
Pregnancy rate	42.9% (6/14)	100.0% (5/5) *

BMI, body mass index; AFC, antral follicle count; FSH, follicle stimulating hormone; LH, luteinizing hormone; E2, estradiol; P4, progesterone; T, testosterone; PRL, prolactin; Gn: gonadotropin; hCG: human chorionic gonadotropin; OPU: oocyte picked-up

Data are presented as mean ± SEM

*$p < 0.05$ compared with GDF-8 < 4.7 ng/ml group

Supplementary Table 4

	GDF-8 decrease from hCG day to OPU day < 1.3 ng/ml (n=8)	GDF-8 decrease from hCG day to OPU day ≥ 1.3ng/ml (n=11)
AGE	28.8±0.9	29.6±1.7
BMI	22.2±1.6	22.6±1.1
Number of AFC	15.7±1.3	12.1±1.5
Basle FSH (mIU/ml)	6.4±0.5	6.4±0.5
Basle LH (mIU/ml)	4.3±0.6	6.1±0.4
Basle E2 (pg/ml)	30.7±6.6	57.1±8.6
Basle P4 (ng/ml)	0.5±0.07	0.6±0.03
Basle T (ng/ml)	0.2±0.04	0.2±0.03
Basle PRL (ng/ml)	23.5±4.3	18.2±2.6
Total Gn days	11.2±0.7	10.7±0.6
Total Gn dose (Ampoules)	1537.0±142.8	1877.5±250.9
Serum E2 on hCG day (pg/ml)	5837.4±581.6	4330.8±641.8
Serum P4 on hCG day (ng/ml)	1.2±0.2	0.8±0.2
Serum E2 on OPU day (pg/ml)	3525.4±694.6	2623.3±414.8
Serum P4 on OPU day (ng/ml)	18.9±3.6	26.5±4.1
P4 increase from hCG day to OPU day (ng/ml)	15.5±4.0	25.7±4.0 *
Number of retrieved oocyte	12.3±1.3	12.8±1.8
Pregnancy rate	25.0% (2/8)	81.8% (9/11) *

BMI, body mass index; AFC, antral follicle count; FSH, follicle stimulating hormone; LH, luteinizing hormone; E2, estradiol; P4, progesterone; T, testosterone; PRL, prolactin; Gn: gonadotropin; hCG: human chorionic gonadotropin; OPU: oocyte picked-up

Data are presented as mean ± SEM

**p* < 0.05 compared with GDF-8 decrease from hCG day to OPU day < 1.3 ng/ml group
